# Feasibility and Operational Limits of a Minimum-Cost Indirect UAV Thermal Sensing Workflow Based on Smartphone-Displayed Infrared Video

**DOI:** 10.3390/s26134259

**Published:** 2026-07-04

**Authors:** Yordan Stoyanov, Atanasi Tashev, Silviya Salapateva, Penko Mitev, Dimitar Yankov, Galya Hristova, Galin Tihanov

**Affiliations:** 1 Department of Transport and Aircraft Equipment and Technologies, Technical University of Sofia, Branch Plovdiv, 25 Tsanko Dyustabanov Street, 4000 Plovdiv, Bulgaria; atanasi.tashev@tu-plovdiv.bg (A.T.); sisisal@tu-plovdiv.bg (S.S.); 2 Center of Competence “Smart Mechatronics, Eco- and Energy Saving Systems and Technologies”, 4000 Plovdiv, Bulgaria; penkomitev@tu-plovdiv.bg (P.M.); d.yankov@tu-plovdiv.bg (D.Y.); 3 Department of Mechanical and Instrument Engineering, Technical University of Sofia, Branch Plovdiv, 25 Tsanko Dyustabanov Street, 4000 Plovdiv, Bulgaria; 4 Department of Electronics, Technical University of Sofia, Branch Plovdiv, 25 Tsanko Dyustabanov Street, 4000 Plovdiv, Bulgaria; 5 Department of Agricultural Engineering, Faculty of Agriculture, Students Campus, Trakia University, 6000 Stara Zagora, Bulgaria; galya.hristova@trakia-uni.bg (G.H.); galin.tihanov@trakia-uni.bg (G.T.)

**Keywords:** UAV, thermal imaging, infrared camera, low-cost sensing, smartphone thermal camera, indirect thermal imaging, payload, motor temperature, battery endurance, display readability, qualitative hotspot indication

## Abstract

**Highlights:**

**What are the main findings?**

- A minimum-cost indirect UAV thermal sensing workflow was experimentally evaluated using a DJI Mini 4K drone, a Servo King9000 smartphone, and a UTi260M smartphone-connected thermal camera.
- The Servo King9000 smartphone acted as the central display and recording interface between the UTi260M thermal camera and the UAV RGB camera.
- The effective added payload was approximately 91 g, corresponding to 36.5% of the UAV mass, and reduced near-ground flight endurance from approximately 25 min to 14 min 40 s.

**What are the implications of the main findings?**

- The proposed workflow is feasible only for short-range qualitative hotspot presence/absence indication under favorable low-glare or nighttime conditions.
- The UAV-recorded output is not direct thermal data, but an RGB recording of a smartphone display showing thermal information; therefore, it is not suitable for quantitative temperature measurement or radiometric thermal mapping.
- Payload mass, suspended-load oscillation, smartphone display readability, motor thermal loading, reduced endurance, and lack of raw radiometric data are the main operational limits.

**Abstract:**

Professional UAV thermal imaging systems are widely used for inspection, environmental monitoring, search and rescue, agriculture, and technical diagnostics. However, their cost limits their use in education, preliminary field screening, rapid prototyping, and low-resource applications. This study evaluates a minimum-cost indirect UAV thermal sensing workflow based on a DJI Mini 4K consumer drone, a lightweight Servo King9000 smartphone, and a UTi260M smartphone-connected infrared thermal camera. In the proposed configuration, the smartphone displayed and recorded the thermal stream, while the onboard RGB camera of the UAV recorded the smartphone-displayed infrared video during flight. The aim was not to develop a radiometric UAV thermal imaging platform, but to determine whether such a low-cost configuration can provide qualitative presence/absence indication of clear thermal hotspots and to identify its operational limits. The system was experimentally assessed under no-payload and payload conditions, daylight and nighttime illumination, and several low-altitude operating heights. Additional motor-region thermal observations were performed using a UTi260T handheld thermal camera under loaded and unloaded operating conditions. The complete UAV–payload configuration had a measured mass of approximately 340 g, corresponding to an effective added payload of 91 g and a payload-to-UAV mass ratio of 36.5%. Payload operation reduced near-ground flight endurance from approximately 25 min to 14 min 40 s. The maximum observed motor-region temperature increased from 24.9 °C under unloaded operation to 42.0 °C under loaded operation, while motor thermal asymmetry increased from 4.8 °C to 7.6 °C. Nighttime and low-glare operation improved the readability of the smartphone-displayed thermal stream, with the most practical usability observed at approximately 10–20 m. The results show that the proposed workflow is feasible only for short-range qualitative thermal screening and clear hotspot presence/absence indication. The UAV-recorded video should not be interpreted as direct thermal data, but as an RGB recording of a smartphone display showing thermal information. Therefore, the workflow is not suitable for quantitative temperature measurement, radiometric thermal mapping, or accurate thermal shape delineation. The main operational limits are payload mass, suspended-load oscillation, display readability, reduced endurance, motor-region thermal loading, sensitivity to payload alignment, and the absence of raw radiometric data. Direct UTi260M smartphone-recorded thermal frames were additionally used for pixel-size-assisted qualitative verification of practical reference thermal targets, including a human-sized target and a vehicle-sized target, at selected low-altitude operating heights.

## 1. Introduction

Unmanned aerial vehicles (UAVs) are widely used for visual inspection, infrastructure monitoring, environmental observation, agricultural assessment, search and rescue, wildlife monitoring, and remote sensing. Their ability to acquire data from elevated viewpoints and difficult-to-access areas makes them valuable tools for rapid field assessment. In addition to conventional RGB imaging, thermal imaging provides information related to heat distribution, thermal anomalies, warm objects, heat losses, and the presence of people or animals under low-light or nighttime conditions.

UAV-based thermal imaging has been applied in a wide range of scientific and practical fields. In wildlife monitoring and search-related applications, thermal UAV data have been used for spatially explicit wildlife occupancy modelling [1], drone-based thermal image tracking for search and rescue [2], nocturnal primate monitoring [3], monitoring of terrestrial mammals [4], optimization of wildlife detection flight paths [5], and evaluation of flight-parameter effects on animal detection in tropical environments [6]. These studies show that thermal UAV performance depends not only on the sensor itself, but also on target size, flight altitude, background conditions, environmental contrast, mission geometry, and data acquisition strategy.

Several studies have also highlighted the operational limitations of thermal UAVs. Burke et al. reported that effective UAV thermal performance in marine and coastal search and rescue depends strongly on target detectability, environmental conditions, acquisition geometry, and mission planning [7]. Nocturnal wildlife monitoring studies have also shown that sensor preparation, operating conditions, and flight strategy influence thermal detectability [4,8]. These findings indicate that UAV thermal systems should be evaluated as complete operational workflows rather than as isolated camera modules.

Thermal UAV imaging is also used in technical, agricultural, and environmental inspection tasks. UAV-based thermal photogrammetry and image processing have been applied for agronomic information extraction [9]. Drone-mounted thermal imaging has been used for peat-fire detection and geolocation [10], urban surface heat monitoring [11], landfill monitoring [12], and mining exploration using UAVs, low-cost thermal cameras, and GIS tools [13]. In agricultural and field environments, UAV thermal systems have also been applied for object detection on agricultural land [14]. These applications demonstrate the broad usefulness of aerial thermal sensing, but they also show that reliable interpretation requires sufficient image quality, stable acquisition geometry, proper flight conditions, and appropriate processing methods.

In building and technical inspection, drone-based thermal approaches have been reviewed as promising tools for integrated building-envelope assessment [15], while UAV thermal cameras have been used for detecting deteriorated photovoltaic modules [16]. These workflows typically rely on directly mounted thermal cameras, stabilized platforms, radiometric acquisition, synchronized telemetry, and dedicated post-processing software. Such systems can provide high-quality thermal outputs, but they also require specialized hardware and higher acquisition costs.

### 1.1. Related Works and Research Gap

Recent UAV sensing research has increasingly focused on multimodal data acquisition, visible–thermal integration, RGB-assisted thermal processing, object detection, and real-time survey workflows [17,18]. These approaches usually rely on advanced hardware, optimized payload integration, stabilized mounting, and more complex processing pipelines. In parallel, UAV deployment under payload variation, wind disturbance, and mechanical uncertainty has motivated research on robust control strategies, including disturbance compensation, nonlinear control, and integrated motor-control approaches [19,20]. This area is relevant to the present study because payload-induced disturbances, center-of-gravity offsets, suspended-load oscillation, and propulsion loading directly influence the usability and safety of lightweight UAV sensing platforms.

Professional thermal UAV workflows normally rely on direct integration of the thermal sensor into the UAV platform. In such systems, the thermal camera is mounted directly on the aircraft, the sensor orientation is controlled, and the acquired data may include radiometric thermal images, synchronized telemetry, and post-processing options. In contrast, very low-cost smartphone-compatible thermal cameras are usually designed for handheld or mobile-phone use rather than for direct UAV integration. Their use in UAV applications therefore introduces additional practical constraints related to smartphone mass, application compatibility, display readability, thermal data format, and mechanical mounting.

Despite the progress in professional UAV thermal imaging, high-quality radiometric thermal UAV systems remain expensive and may be inaccessible for educational demonstrations, preliminary feasibility studies, rapid prototyping, and low-resource field screening. This creates interest in alternative low-cost configurations that can provide basic thermal information without attempting to replace professional radiometric UAV platforms. Smartphone-compatible thermal cameras represent one possible low-cost solution because they are compact, relatively inexpensive, and capable of displaying or recording thermal video through mobile applications.

However, integrating a smartphone-connected thermal camera with a lightweight consumer UAV is technically challenging. The added mass of the smartphone and thermal camera can significantly affect flight endurance, motor loading, center-of-gravity position, suspended-load stability, and safety margins. In addition, when the UAV does not carry a dedicated thermal camera, one possible workaround is to record the smartphone display using the onboard RGB camera of the UAV. This creates an indirect acquisition chain in which the thermal camera generates the primary thermal stream, the smartphone displays the stream, and the UAV RGB camera records the displayed thermal image during flight.

This indirect approach is fundamentally different from professional UAV thermal imaging. The UAV-recorded video is not direct thermal data, but an RGB video of a smartphone display showing thermal information. As a result, the recorded output is affected by display brightness, ambient illumination, reflections, camera exposure, focus, display size in the frame, screen refresh effects, compression, and payload motion. Therefore, such a workflow cannot be used for quantitative temperature measurement, radiometric thermal mapping, or accurate shape delineation. Its potential value is limited to qualitative indication of the presence or absence of clearly distinguishable thermal hotspots under favorable operating conditions.

Previous studies have addressed several adjacent aspects of the workflow investigated in the present work. Vasterling and Meyer discussed the opportunities and practical constraints of UAV-borne thermal imaging, including sensor integration, acquisition geometry, and operational limitations [21]. Bendig et al. introduced a low-cost mini-UAV platform for thermal and multispectral imaging, showing that low-cost UAV-based thermal sensing is feasible but strongly constrained by payload capacity, sensor mass, and acquisition conditions [22]. In addition, smartphone-connected thermal cameras have been evaluated as accessible low-cost sensing tools; for example, Petrie et al. assessed a smartphone-based thermal camera system for grapevine water-status estimation, demonstrating the practical value of smartphones as acquisition and processing interfaces in low-cost thermal sensing workflows [23]. However, such systems are normally used as handheld or ground-based tools rather than as suspended UAV payloads.

More recent UAV thermal studies have emphasized that quantitative temperature estimation requires controlled calibration, stable acquisition geometry, and careful treatment of environmental and sensor-related effects. Han et al. showed that temperature-controlled ground references can substantially improve the accuracy of UAV-based thermal infrared imagery [24], while Wan et al. demonstrated that ambient conditions, flight altitude, and intrinsic uncooled-camera dynamics can significantly affect UAV-measured temperatures [25]. Similar feasibility-oriented UAV thermal studies in vineyards further confirm that aerial thermal workflows must be interpreted together with their experimental conditions and measurement limitations [26]. These requirements are not satisfied by the indirect display-recording workflow evaluated in the present study. Therefore, the proposed system should be interpreted only as a qualitative hotspot presence/absence indication method, not as a radiometric UAV thermal measurement platform. Furthermore, studies on suspended-cable payload transportation with quadrotors show that suspended loads introduce pendulum-like motion, cable tension effects, center-of-gravity variation, and control-related disturbances [27], which are directly relevant to the cord-suspended smartphone–thermal camera module used in this work.

The research gap addressed in this study is the lack of experimental characterization of minimum-cost indirect UAV thermal workflows based on smartphone-displayed infrared video. While professional UAV thermal platforms, radiometric aerial thermography, and advanced visible–thermal fusion methods have been widely investigated, less attention has been given to configurations in which a smartphone-connected thermal camera is operated by a lightweight smartphone and the displayed thermal stream is recorded indirectly by the UAV RGB camera. This gap is important because such a configuration introduces limitations that are not present in direct radiometric UAV thermal systems, including smartphone payload mass, suspended-load oscillation, display readability, RGB camera exposure and focus, screen reflections, lack of raw radiometric data, reduced endurance, motor loading, and safety margins for lightweight sub-250 g UAV operation. Therefore, the combined operational effect of these factors requires experimental evaluation before such a workflow can be considered suitable even for preliminary qualitative screening.

The present study evaluates a minimum-cost indirect UAV thermal sensing workflow based on a DJI Mini 4K consumer drone, a Servo King9000 lightweight smartphone, and a UTi260M smartphone-connected infrared thermal camera. The smartphone–thermal camera module was suspended below the UAV using an ultralight cord-based mount, while the onboard RGB camera recorded the displayed thermal video stream during flight. Additional motor-region thermal observations were performed using a UTi260T handheld thermal camera in order to assess the effect of unloaded and loaded operation on propulsion thermal loading.

### 1.2. Objective and Contributions

The objective of this study is to experimentally evaluate the feasibility and operational limits of a minimum-cost UAV–smartphone–thermal camera workflow under exploratory field conditions. The system is not intended as a radiometric UAV thermal imaging platform and is not proposed as a substitute for professional UAV thermography. Instead, it is evaluated as a qualitative, short-range, low-altitude screening method for visual presence/absence indication of clear thermal hotspots. The analysis focuses on payload mass, relative UAV loading, suspended-load oscillation, display readability, operating height, illumination conditions, endurance reduction, motor-region thermal response under loaded and unloaded operation, operational warnings, and the practical limits of indirect non-radiometric thermal visualization.

The main contributions of this study are as follows:

1. A minimum-cost indirect UAV thermal sensing workflow is experimentally evaluated using a consumer drone, a lightweight smartphone, and a smartphone-connected LWIR thermal camera.
2. The role of the smartphone as the central display and recording interface between the thermal camera and the UAV RGB camera is clarified.
3. The payload mass and payload-to-UAV mass ratio are quantified, and their effects on endurance, stability, and operational safety are assessed.
4. The influence of the suspended smartphone–thermal camera module on flight endurance, payload oscillation, motor thermal loading, and warning occurrence is documented.
5. The usability of smartphone-displayed thermal video recorded indirectly by the UAV RGB camera is compared under daylight and nighttime conditions.
6. Direct thermal-camera frames and UAV-recorded display frames are compared to clarify the difference between primary thermal information and indirect display-based visualization.
7. The operational boundary of the proposed workflow is defined, including its limitation to qualitative hotspot presence/absence indication and its unsuitability for quantitative temperature measurement or radiometric thermal mapping.

The novelty of this work is not the development of a professional UAV thermal imaging system, but the experimental characterization of an intentionally minimal and low-cost indirect thermal sensing configuration operating close to the practical limits of a lightweight consumer UAV. The study provides an engineering assessment of what can and cannot be achieved when a smartphone-connected thermal camera is integrated with a small UAV without a dedicated thermal payload, gimbal stabilization, raw radiometric export, or onboard thermal-video transmission.

## 2. Materials and Methods

### 2.1. Study Design and Experimental Workflow

This study was designed as an experimental feasibility and operational-limit assessment of a minimum-cost indirect UAV thermal sensing workflow. The objective was to evaluate whether a lightweight consumer UAV could carry a smartphone-connected thermal camera module and whether the smartphone-displayed infrared video could be recorded by the UAV onboard RGB camera during flight.

The study did not aim to develop a professional radiometric UAV thermal imaging platform. Instead, it focused on the practical boundary at which a low-cost UAV, a lightweight smartphone, and a smartphone-connected thermal camera can still provide qualitative thermal information. The intended use of the workflow was limited to short-range preliminary screening and presence/absence indication of clear thermal hotspots.

The experimental workflow consisted of the following steps: system assembly, payload mass measurement, evaluation of the suspended mounting geometry, no-payload baseline flight testing, payload flight testing under daylight and nighttime conditions, direct recording of the thermal stream by the smartphone-connected thermal camera, indirect recording of the smartphone-displayed thermal stream by the UAV RGB camera, motor-region thermal observation under unloaded and loaded operating conditions, and identification of the main operational limits.

The workflow was intentionally based on commercially available low-cost components. This allowed the study to evaluate the practical limitations of an extremely low-cost configuration rather than the performance of an optimized professional UAV thermal platform.

**Figure 1:**
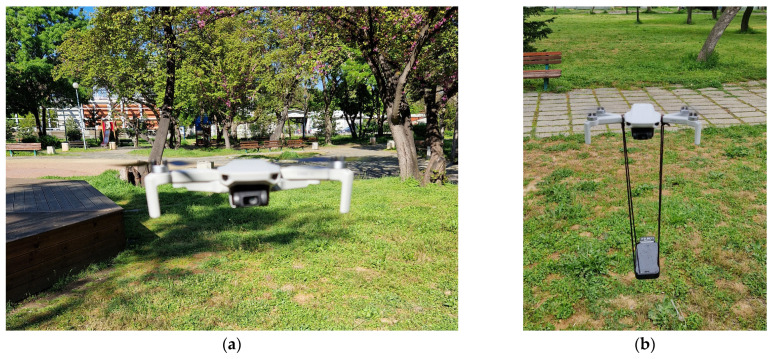
Experimental system and workflow used in the study: (**a**) DJI Mini 4K during baseline no-payload operation; (**b**) DJI Mini 4K with the suspended Servo King9000 smartphone and attached UTi260M thermal camera.

The experimental UAV configuration and the general workflow used in the study are shown in Figure 1.

### 2.2. System Architecture and Role of the Smartphone

The proposed system consisted of five main hardware elements: a consumer UAV platform, a lightweight smartphone used as the thermal display and recording unit, a smartphone-connected thermal camera, an auxiliary smartphone used for UAV control and monitoring, and a handheld thermal camera used for post-flight motor inspection.

The UAV platform was a DJI Mini 4K consumer drone. The airborne thermal module consisted of a Servo King9000 lightweight Android smartphone connected to a UTi260M smartphone-compatible infrared thermal camera. The UTi260M generated the primary thermal stream, while the Servo King9000 operated the thermal camera application, displayed the live infrared video, and recorded the direct thermal output locally.

In the proposed workflow, the Servo King9000 smartphone was not only an added payload, but the central display and recording interface between the UTi260M thermal camera and the UAV RGB camera. Without the smartphone, the UTi260M thermal stream could not be operated, visualized, recorded locally, or indirectly captured by the UAV onboard camera. Therefore, the feasibility of the whole workflow depended strongly on the smartphone characteristics, including its mass, display size, display brightness, application compatibility, battery capacity, and mechanical integration with the thermal camera.

During flight, the onboard RGB camera of the DJI Mini 4K recorded the smartphone display. Therefore, the UAV did not directly acquire thermal data. Instead, it recorded an RGB video of the smartphone-displayed thermal stream. This created an indirect display-based thermal sensing workflow.

A Ulefone Armor 27T smartphone was used for UAV control, live monitoring, telemetry visualization, and screen recording. A UTi260T handheld thermal camera was used to observe the thermal state of the UAV motor regions under unloaded and loaded operating conditions.

The system architecture was based on two visual data channels:

1. direct thermal-camera output recorded by the Servo King9000 smartphone from the UTi260M thermal camera;
2. indirect UAV RGB video showing the smartphone-displayed thermal stream during flight.

The first channel was treated as the primary thermal reference. The second channel was treated as a feasibility indicator for indirect UAV-based thermal visualization. This distinction is essential because the UAV-recorded frames are not radiometric thermal images, but RGB recordings of a display showing thermal information.

### 2.3. Technical Characteristics of the Experimental Components

The main components used in the experimental workflow are summarized in Table 1. The table includes the role of each device, its relevant technical characteristics, and its function within the proposed indirect sensing system.

**Figure 2:**
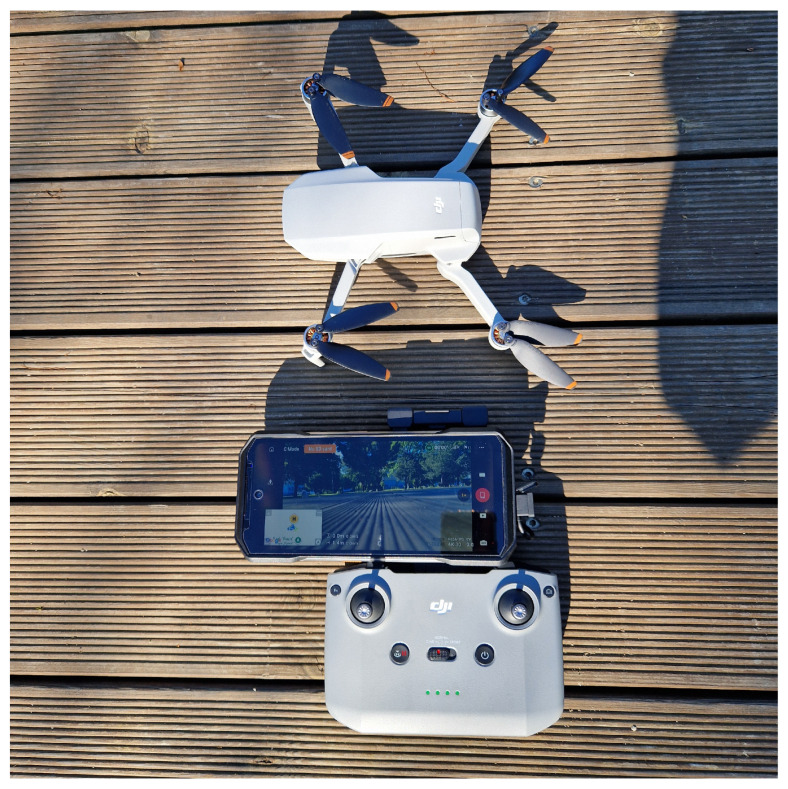
Ground control and monitoring configuration of the experimental UAV thermal sensing workflow. The DJI Mini 4K platform was operated using the remote controller and the Ulefone Armor 27T smartphone, which provided live UAV camera monitoring, telemetry visualization, and screen recording during the field tests.

The ground control and monitoring configuration used during the field tests is shown in Figure 2.

### 2.4. Payload Mass and Relative Loading

The masses of the UAV and payload components were measured using a digital scale. The bare DJI Mini 4K platform had a measured mass of approximately 249 g. The complete UAV–payload configuration had a measured mass of approximately 340 g. Therefore, the effective added payload was calculated as the difference between the complete UAV–payload configuration and the UAV alone.

**Figure 3:**
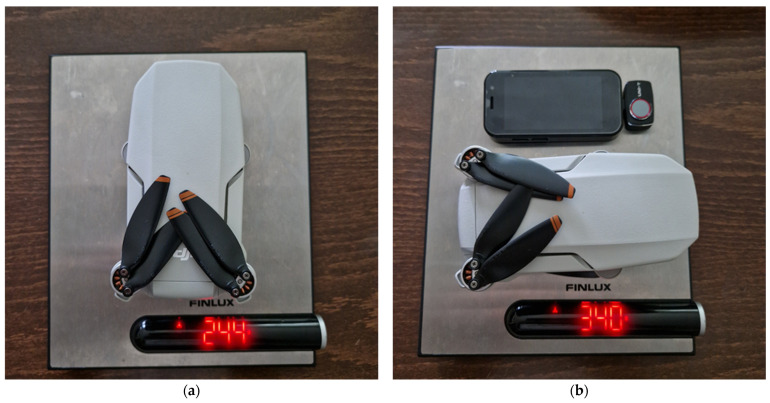
Payload characterization and suspension geometry of the experimental UAV thermal sensing configuration: (**a**) DJI Mini 4K platform mass; (**b**) Servo King900 smartphone with attached UTi260M thermal camera.

The measured masses and payload configuration are shown in Figure 3, and the corresponding values are summarized in Table 2.

The effective added payload was calculated as:

(1) $$m_{payload}=m_{total}-m_{UAV}$$

where $m_{payload}$ is the effective payload mass, $m_{total}$ is the measured mass of the complete UAV–payload configuration, and $m_{UAV}$ is the measured mass of the UAV without the suspended module.

Using the measured values:

$$m_{payload}=340-249=91g.$$

The relative payload ratio was calculated as:

(2) $$PR=\frac{m_{payload}}{m_{UAV}}100$$

$$PR=\frac{91}{249}100=36.5\%.$$

Thus, the effective payload represented approximately 36.5% of the UAV body mass. This is a substantial value for a lightweight consumer UAV platform and was expected to affect flight stability, motor loading, battery endurance, and operational safety margins.

### 2.5. Payload Suspension and Camera–Display Geometry

The Servo King9000 smartphone with the attached UTi260M thermal camera was suspended below the UAV using an ultralight cord-based mounting configuration. The approximate distance between the UAV onboard RGB camera and the smartphone display was 0.30 m, as shown in Figure 2.

This distance was selected as a practical compromise. At shorter distances, the smartphone display occupied a larger portion of the UAV camera frame, but the onboard RGB camera tended to lose focus. At longer distances, the display was easier to focus but became smaller in the recorded frame, reducing the readability of the displayed thermal stream. The 0.30 m distance was therefore selected as a compromise between display visibility and focus stability.

The cord-based suspension reduced the mass of the mounting system, but introduced pendulum-like motion. This motion was particularly visible during take-off, vertical movement, hover correction, and small attitude changes of the UAV. In addition, perfect alignment of the smartphone–thermal camera module with the UAV centerline was difficult to achieve. Even a small horizontal offset between the payload center of mass and the UAV center of gravity could generate a destabilizing moment.

The payload weight force was calculated as:

(3) $$F=m_{payload}g,$$

where F is the payload weight force, $m_{payload}$ is the effective payload mass and $g=9.81\;m/s^{2}$. For the effective payload mass of 91 g:

F=0.091·9.81=0.893 N.

This value corrects the payload force calculation and shows that the force is not large in absolute terms. However, because the load was suspended below the UAV and could be slightly offset from the centerline, it introduced an additional moment.

The destabilizing moment caused by an offset suspended payload can be expressed as:

(4) M=Fd,

where M is the moment caused by the suspended payload, F is the payload weight force, and d is the horizontal offset between the payload center of mass and the UAV center of gravity.

Although the absolute payload force was below 1 N, its suspended position, possible horizontal offset, and oscillatory motion increased the control demand on the UAV propulsion system. The payload was also exposed to rotor downwash, which interacted with the smartphone body and the thermal camera module. This airflow interaction contributed to payload oscillation and reduced the stability of the smartphone-displayed thermal stream recorded by the UAV RGB camera.

### 2.6. Direct-Mounting Alternative and Reason for the Suspended Configuration

A direct rigid attachment of the smartphone–thermal camera module to the UAV could reduce payload oscillation and improve mechanical stability. However, in such a configuration, the onboard RGB camera of the UAV would no longer record the smartphone display. The thermal information would remain available mainly as a local recording on the smartphone, unless an additional wireless streaming or data-transmission method was used.

Therefore, the suspended display-facing configuration was selected in this study specifically to evaluate the feasibility and limitations of indirect in-flight thermal visualization through the existing UAV RGB camera. This configuration is mechanically less stable than a rigid mount, but it enables the UAV camera to record the smartphone-displayed thermal stream without modifying the UAV hardware.

A more rigid mount, including a lightweight composite or carbon-fiber support, could reduce payload oscillation and improve the stability of the smartphone-displayed thermal stream in future configurations. However, such a solution was not implemented in the present study because the objective was to evaluate a minimum-cost and mass-minimized configuration based on readily available components. Introducing a specialized rigid composite mount would increase system cost, change the mechanical design, and move the system away from the intended low-cost proof-of-concept boundary.

The suspended configuration should therefore be interpreted as a boundary-condition proof-of-concept rather than an optimized payload-mounting solution.

### 2.7. Experimental Flight Conditions and Measurement Campaigns

The experimental tests were performed as an exploratory field assessment of the proposed indirect UAV thermal visualization workflow. The purpose was to identify the main practical limits of the configuration rather than to perform a statistically validated detection study. The tests included no-payload baseline operation, payload hover, daylight and nighttime display recording, altitude-dependent usability assessment, direct thermal-camera recording, indirect UAV recording of the smartphone display, motor-region thermal observation under unloaded and loaded operating conditions, and observation of UAV warning messages.

The study used one UAV platform, one smartphone–thermal camera payload configuration, and selected representative field trials under the available operating conditions. Environmental conditions such as wind, illumination, ambient temperature, and battery state were not controlled using laboratory instrumentation. Instead, they were treated as practical field variables and are reported as operational limitations of the study.

The experimental campaigns and measurement scenarios are summarized in Table 3.

No-payload baseline flights were used to document the normal behavior of the UAV platform without the suspended thermal module. Payload flights were then performed with the Servo King9000 + UTi260M module suspended below the UAV in order to evaluate lifting capability, hover stability, endurance reduction, display readability, and operational warnings.

Daylight tests were used to assess the influence of sunlight, glare, and reflections on the readability of the smartphone-displayed thermal stream in the UAV RGB recording. Nighttime tests were used to assess whether reduced ambient illumination improved display readability and visual distinguishability of clear thermal hotspots.

Altitude-dependent usability was assessed at several selected low-altitude operating heights, including approximately 5–7 m, 10–15 m, 20 m, and 24–25 m. A higher altitude of approximately 120 m was used only as a boundary or stress test and was not considered a recommended normal operating mode. Height values were extracted from the UAV telemetry overlay visible in the recorded RGB frames.

Flight endurance was evaluated for no-payload and payload configurations. The no-payload near-ground hover endurance was approximately 25 min, while the payload configuration achieved approximately 14 min 40 s under non-ideal stabilization conditions. A more intensive payload flight profile, including ascent to higher altitude, resulted in an endurance of approximately 13 min.

Because the study was exploratory, the number of repeated trials was limited and environmental variables were not controlled using calibrated field instruments. Therefore, the results are interpreted as practical feasibility and operational-limit observations rather than statistically validated UAV thermal detection performance.

### 2.8. Thermal and RGB Data Acquisition

The UTi260M thermal camera produced the primary thermal stream. This stream was displayed and recorded on the Servo King9000 smartphone through the thermal camera application. The UAV onboard RGB camera recorded the smartphone display during flight, producing a secondary video channel of the displayed thermal information.

Two types of visual data were therefore analyzed:

1. direct thermal frames recorded by the Servo King9000 smartphone from the UTi260M thermal camera;
2. indirect UAV RGB frames showing the smartphone-displayed thermal stream during flight.

The direct thermal frames were treated as the primary thermal reference. The UAV RGB recordings were treated as secondary evidence of the feasibility and limitations of indirect display-based thermal visualization.

This distinction is essential. The UAV RGB frames do not represent direct radiometric UAV thermal data. They represent an RGB recording of a display showing thermal information. Therefore, the indirect UAV-recorded output is affected by display brightness, camera focus, exposure control, screen reflections, compression, display size, suspended-load motion, and acquisition geometry.

For this reason, the indirect UAV-recorded video was interpreted only as a qualitative visualization channel. It was used to assess whether clear thermal hotspots could be visually indicated during flight, but it was not used for quantitative temperature measurement, radiometric mapping, or accurate thermal shape delineation.

### 2.9. Thermal Data Format and Radiometric Limitations

The thermal data format represents one of the main methodological limitations of the proposed workflow. The UTi260M smartphone-connected thermal camera operates through a dedicated mobile application and provides thermal images and videos at the application level. In the experimental configuration used in this study, direct access to raw radiometric matrices, frame-by-frame temperature arrays, and sensor-level thermal data was not available.

Consequently, the thermal information analyzed in this work was derived from application-generated thermal images, videos, displayed temperature values, exported frames, and UAV-recorded RGB videos of the smartphone display rather than from raw radiometric datasets. This limitation prevents precise radiometric post-processing and restricts the analysis to qualitative thermal interpretation and practical image-usability assessment.

The UAV-recorded output represents an RGB recording of a smartphone display showing thermal information. It is therefore affected by the thermal camera, the smartphone display, the UAV RGB camera, screen brightness, ambient illumination, camera exposure, focus, suspended-load motion, and video compression. For this reason, it cannot be used for quantitative temperature measurement, radiometric thermal mapping, or accurate thermal shape delineation.

The UTi260T handheld thermal camera was used primarily for post-flight motor thermal inspection. Although the camera provides temperature information, the measurements obtained in this study should be interpreted as practical engineering observations rather than traceable laboratory-grade thermometric measurements.

Therefore, the proposed workflow should not be interpreted as a radiometric UAV thermal imaging system. Instead, it should be regarded as an indirect thermal visualization workflow intended for qualitative hotspot presence/absence indication and preliminary thermal screening.

### 2.10. Pixel-Size-Assisted Verification of Practical Thermal Reference Targets

To strengthen the field-based interpretation of the proposed workflow, selected direct UTi260M smartphone-recorded thermal frames were additionally used for qualitative target-size verification. The purpose of this step was not to develop an automatic human-detection algorithm or a validated target-classification system. Instead, the analysis was used to check whether clearly visible thermal hotspots were compatible with practical reference targets of known type, namely a human-sized target and a vehicle-sized target.

Only the direct UTi260M thermal frames recorded on the Servo King9000 smartphone were used for this pixel-size-assisted check. The UAV-recorded RGB video of the smartphone display was not used for pixel-size estimation because it represents an indirect optical recording of a screen and is affected by display brightness, camera exposure, focus, compression, screen size in the frame, and payload motion. The UAV telemetry overlay was used only to assign the approximate operating height corresponding to the selected thermal frames.

For each selected thermal frame, the visible hotspot corresponding to the reference target was isolated manually or semi-manually from the surrounding background. The apparent target footprint was then described using the bounding-box dimensions in pixels, the approximate number of hotspot pixels, and the target-to-background thermal or grayscale contrast. Because raw radiometric matrices were not available, these values were interpreted only as relative indicators of target visibility and apparent size, not as calibrated radiometric measurements.

If the camera field of view and flight height are known, the approximate ground-projected frame dimensions can be estimated using Ws = 2H · tan(FOVh/2) and Ls = 2H tan · (FOVv/2), where Ws and Ls are the estimated ground-projected frame width and length, respectively; H is the approximate UAV operating height; and FOVh and FOVv are the horizontal and vertical fields of view of the thermal camera.

The corresponding ground sampling distances are GSDx = Ws/Nx and GSDy = Ls/Ny, where Nx and Ny are the horizontal and vertical pixel dimensions of the thermal frame.

The approximate physical dimensions of the thermal target can then be estimated from its bounding box using Wt = wbbox · GSDx and Lt = hbbox · GSDy, where Wt and Lt are the estimated target-footprint width and length, respectively, and wbbox and hbbox are the bounding-box width and height in pixels.

This procedure was used only as a qualitative consistency check. A detected hotspot was interpreted as compatible with a human-sized or vehicle-sized target only when its apparent pixel footprint, estimated size range, and target-to-background contrast were consistent with the expected field target.

### 2.11. Qualitative Image Usability Assessment

Because raw radiometric matrices were unavailable and because the UAV-recorded frames were RGB recordings of a smartphone display, image assessment was performed qualitatively. The purpose was not to quantify thermal accuracy, but to determine whether the displayed thermal stream remained visually usable under the tested operating conditions.

The qualitative assessment considered the following factors:

1. Visibility of the smartphone display in the UAV RGB frame;
2. Readability of the thermal stream shown on the smartphone display;
3. Presence of glare, reflections, or overexposure;
4. Apparent stability of the displayed thermal image during suspended-payload motion;
5. Visual distinguishability of clear thermal hotspots from the surrounding background;
6. Comparison between the direct UTi260M thermal recording and the indirect UAV-recorded display frame.

Image usability was classified descriptively as good, limited, or not usable. A frame was considered good when the smartphone display was clearly visible and the thermal hotspot was visually distinguishable. A frame was considered limited when the display was visible but affected by glare, overexposure, reduced size, or motion. A frame was considered not usable when the displayed thermal stream could not be interpreted reliably from the UAV RGB recording.

### 2.12. Flight Endurance and Payload Penalty

Flight endurance was evaluated under no-payload and payload conditions. The relative reduction in flight endurance was calculated using the following relationship:

(5) $$R_{t}=\frac{t_{baseline}-t_{payload}}{t_{baseline}}\cdot 100$$

where $R_{t}$ is the relative endurance reduction, $t_{baseline}$ is the no-payload flight time, and $t_{payload}$ is the payload flight time.

For the near-ground hover case:

$$t_{baseline}=25\;min$$

$$t_{payload}=14.67\;min$$

then:

$$R_{t}=\frac{25-14.67}{25}\cdot 100\approx 41.3\%$$

For the more intensive payload flight profile, including ascent to higher altitude, the payload endurance was approximately 13 min. Therefore:

$$t_{payload,intensive}=13\;min$$

$$R_{t}=\frac{25-13}{25}\cdot 100\approx 48\%$$

Because complete start–end battery percentage records were not available for all test segments, the present analysis focused mainly on total endurance and relative endurance reduction. The endurance results should therefore be interpreted as practical operational indicators rather than controlled battery-performance measurements.

### 2.13. Motor-Region Thermal Observation Under Loaded and Unloaded Operation

Motor thermal loading was evaluated using UTi260T thermal images acquired after selected no-payload and payload flights. The purpose of this analysis was to compare post-flight motor-region temperatures under unloaded and loaded operating conditions.

The motor temperature difference between payload and no-payload operation was estimated as:

(6) $$\Delta T_{motor}=T_{motor,payload}-T_{motor,no-payload}$$

When ambient or background temperature was available, the normalized motor temperature rise was calculated as:

(7) $$\Delta T_{motor}=T_{motor}-T_{ambinet}$$

The maximum motor temperature was defined as:

(8) $$T_{max}=max\;(T_{M1},T_{M2},T_{M3}.T_{M4})$$

Motor thermal asymmetry was defined as:

(9) $$TA=T_{max}-T_{min}$$

where $T_{max}$ and $T_{min}$ are the maximum and minimum motor-region temperatures after flight. This parameter was used to evaluate whether the payload caused uneven propulsion loading.

It should be emphasized that these measurements were obtained after landing and therefore do not represent real-time in-flight motor temperatures. The measured values should be interpreted as conservative indicators of payload-induced thermal loading because partial cooling occurs during descent, landing, and the time interval before thermal image acquisition.

Continuous thermal imaging of the motors during take-off or free flight was not performed. Such a measurement would require either a fixed UAV test configuration or a separate synchronized thermal recording system with stable line of sight to the motors. Fixing the UAV during propeller operation would alter the natural flight-control response and would not reproduce the same loading conditions as free flight. In addition, close-range thermal imaging during take-off would introduce safety and optical-access limitations due to propeller motion and rotor downwash. Real-time motor telemetry, ESC temperature logging, or embedded temperature sensors are therefore considered necessary for future in-flight motor thermal validation.

### 2.14. Descriptive Operational Interpretation

The dataset used in this study was intentionally limited and was not designed for rigorous statistical validation. Therefore, the operational relationships presented in this work were interpreted as descriptive and exploratory observations rather than statistically confirmed correlations.

The following relationships were examined descriptively:

1. Payload condition versus endurance reduction;
2. Payload condition versus post-flight motor thermal loading;
3. Payload condition versus warning occurrence;
4. Illumination condition versus display readability;
5. Operating height versus display usability;
6. Payload alignment versus propulsion asymmetry.

Because of the limited number of observations, confidence intervals, inferential statistics, predictive modelling, and formal correlation analysis were not considered appropriate. The purpose of the analysis was to identify practical trends and operational limits that may guide future investigations.

Consequently, all reported relationships should be interpreted as preliminary indications observed under the tested conditions and not as statistically validated engineering laws.

## 3. Results

### 3.1. Basic Feasibility of the Indirect UAV Thermal Workflow

The first experimental result was the successful operation of the UTi260M smartphone-connected thermal camera with the lightweight Servo King9000 smartphone. This combination formed a functional thermal display and recording module with a mass below 100 g. The complete UAV-payload configuration had a measured mass of approximately 340 g and was successfully lifted by the DJI Mini 4K platform.

This confirmed the basic feasibility of the proposed low-cost indirect UAV thermal sensing workflow. The system was able to acquire a primary thermal stream through the smartphone application and simultaneously allow the UAV onboard RGB camera to record the smartphone-displayed thermal image during flight.

Using Equations (1) and (2), the effective added payload was calculated as 91 g, corresponding to a payload-to-UAV mass ratio of 36.5%. These calculated values were used to interpret the subsequent endurance, motor-loading, and stability results.

### 3.2. Payload Effect on Flight Endurance

The no-payload UAV baseline flight was completed without abnormal behavior and achieved approximately 25 min of near-ground hover endurance. With the suspended Servo King9000 + UTi260M payload, the near-ground hover endurance decreased to approximately 14 min 40 s. Under a more intensive flight profile involving active stabilization and ascent to 120 m, the payload endurance decreased further to approximately 13 min. The measured endurance values and relative reductions are summarized in Table 4.

The relative endurance reductions reported in Table 4 were calculated using Equation (5), with the no-payload hover endurance used as the baseline reference.

According to Equation (5), the payload-hover endurance reduction was approximately 41.3%, while the intensive payload-flight profile resulted in an endurance reduction of approximately 48.0%.

The results show that the payload configuration significantly reduced flight endurance. The 14 min 40 s value should be interpreted as a near-ground hover reference rather than a full mission endurance value. More demanding flight profiles involving horizontal displacement, ascent, descent, wind compensation, and payload oscillation damping further increased propulsion demand and reduced usable flight duration.

### 3.3. Payload Effect on Motor Thermal Loading

Thermal observations showed a clear increase in motor-region temperature under loaded operation. In the unloaded condition, the maximum observed motor-region temperature was 24.9 °C. Under loaded operation with the suspended smartphone–thermal camera module, the maximum observed motor-region temperature increased to 42.2 °C. The corresponding motor thermal asymmetry increased from 4.8 °C under unloaded operation to 7.6 °C under loaded operation. These values indicate increased propulsion thermal loading caused by the added payload; however, they should be interpreted as post-operation motor-region indicators rather than real-time in-flight motor temperature measurements. The representative motor thermal loading comparison under no-payload and payload conditions is summarized in Table 5.

The maximum motor-region temperatures were interpreted using Equation (8), while the loaded–unloaded motor-temperature differences were assessed using Equation (6).

Baseline flights without payload were completed without instability, propulsion warnings, or abnormal thermal loading. In contrast, the suspended payload configuration produced increased motor temperatures and occasional warnings. This indicates that the observed limitations were payload-induced rather than caused by a defective UAV platform.

### 3.4. Influence of Payload Orientation and Center-of-Gravity Offset

Repeated tests showed that payload orientation was a critical parameter. A slight forward inclination of the smartphone-thermal camera module toward the front motors caused increased thermal loading and persistent warning messages during flight. This confirmed that the system is highly sensitive not only to payload mass, but also to payload alignment and center-of-gravity offset.

The effect can be explained by the moment generated by the suspended load. When the center of mass of the module is shifted forward, the propulsion system must continuously compensate for the resulting moment. This leads to asymmetric motor loading, increased heating, and reduced operational robustness.

### 3.5. Influence of Illumination and Display Brightness

Daylight operation was limited mainly by display glare and reflections. Under direct sunlight, the smartphone-displayed thermal stream was more difficult to interpret from the UAV RGB recording. In contrast, nighttime and low-glare conditions improved the readability of the smartphone display in the UAV-recorded video.

In dark conditions, reducing the smartphone display brightness to approximately 50% improved the balance between display visibility and overexposure. Maximum brightness was not always optimal during nighttime operation because it could overexpose parts of the smartphone display in the UAV RGB recording.

**Figure 4:**
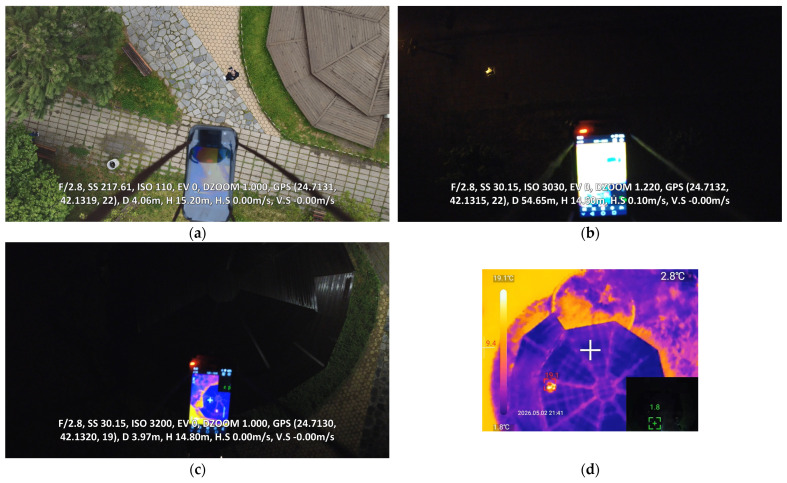
Influence of illumination and smartphone display brightness on indirect UAV thermal visualization: (**a**) daylight UAV RGB recording, where the smartphone-displayed thermal stream is poorly readable due to ambient illumination and reflections; (**b**) nighttime UAV RGB recording with high display brightness, resulting in partial overexposure of the display; (**c**) nighttime UAV RGB recording with reduced display brightness, showing improved readability of the thermal stream despite the suspended payload configuration; (**d**) direct thermal frame recorded by the Servo King9000 smartphone with the UTi260M thermal camera, used as the primary thermal reference for cross-checking the UAV-recorded display frames.

Representative frames illustrating the influence of illumination and display brightness are shown in Figure 4. During daylight operation, the smartphone-displayed thermal stream was difficult to interpret from the UAV RGB recording because of ambient illumination, reflections, and reduced display readability. During nighttime operation, display readability improved; however, excessive smartphone brightness caused partial overexposure in the UAV RGB recording. Reducing the smartphone brightness improved the balance between display visibility and overexposure.

The direct UTi260M thermal recording was used as the primary reference to verify that the thermal content observed indirectly by the UAV camera corresponded to the smartphone-displayed thermal stream.

The results indicate that illumination primarily affected image usability rather than flight endurance. Nighttime operation improved the readability of the displayed thermal stream, but did not reduce the energy penalty caused by the suspended payload.

### 3.6. Altitude Range and Image Usability

Nighttime tests showed that the smartphone-displayed thermal stream remained visible to the UAV RGB camera at several low-altitude operating heights. Usable indirect visualization was observed at approximately 5–7 m, 10–15 m, 20 m, and up to approximately 24–25 m. The most practical image usability was observed in the 10–20 m range. At higher altitudes, the smartphone display remained visible but became smaller in the UAV RGB frame and more difficult to interpret.

The observed image usability at different operating heights is summarized in Table 6.

**Figure 5:**
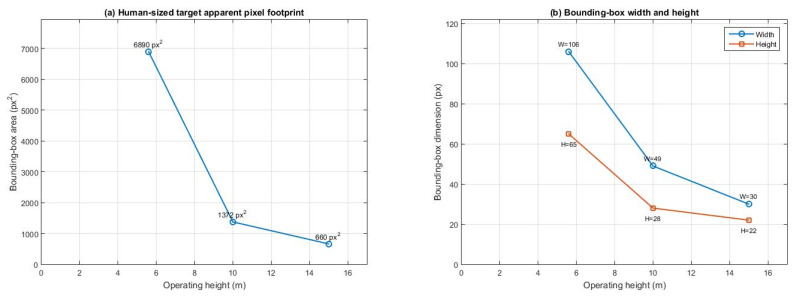
Apparent pixel footprint of a human-sized thermal target at different low-altitude operating heights: (**a**) bounding-box area of the target in direct UTi260M smartphone-recorded thermal frames; (**b**) bounding-box width and height as a function of operating height. The bounding-box footprint decreased from 6890 px^2^ at 5.6 m to 1372 px^2^ at 10 m and 660 px^2^ at 15 m, confirming that the apparent thermal representation of the target decreases with increasing height. The UAV RGB video was used only to assign the approximate operating height and was not used for pixel-size estimation.

To further support the qualitative height-dependent usability assessment, a pixel-size-assisted verification was performed using a human-sized thermal reference target. The apparent target footprint at different operating heights is presented in Figure 5.

The values in Table 6 should not be interpreted as quantitative detection thresholds. They describe practical image usability and visual readability of the smartphone-displayed thermal stream under the tested conditions. Similarly, the pixel-footprint values in Figure 5 should be interpreted only as a qualitative target-size consistency check, not as a validated human-detection threshold or a statistical detection probability. 

**Figure 6:**
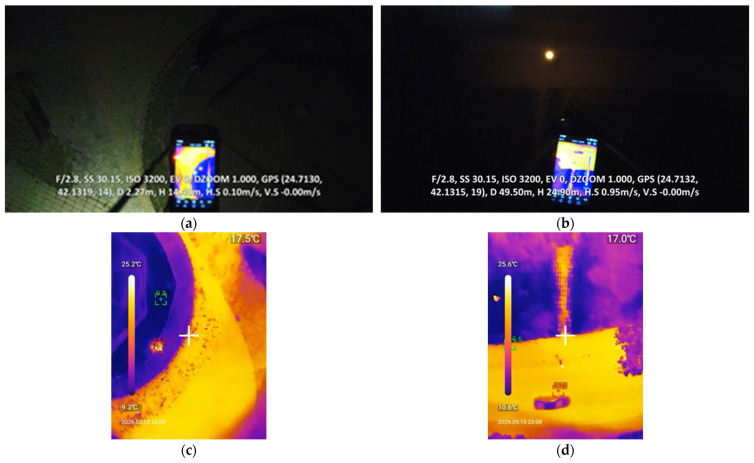
Representative dual-channel comparison between direct thermal-camera output and UAV-recorded smartphone display: (**a**,**c**) direct thermal frames recorded by the Servo King9000 smartphone with the UTi260M thermal camera; (**b**,**d**) corresponding or approximately corresponding UAV RGB frames showing the smartphone-displayed thermal stream during flight. The comparison demonstrates that the direct thermal stream provides clearer thermal information, while the UAV-recorded display remains usable for preliminary monitoring despite payload oscillation, reduced sharpness, display-related effects, and indirect optical acquisition.

In addition to the qualitative usability assessment, a short descriptive entropy check was performed for six consecutive smartphone-displayed thermal frames from the scene shown in Figure 6c. Each selected frame was converted to an 8-bit grayscale image, and Shannon entropy was calculated from the same region of interest in all frames. The temperature scale, crosshair, application overlays, and interface elements were excluded from the analyzed region as far as possible. The entropy values were used only as relative image-information indicators and were not interpreted as radiometric measurements, detection accuracy, or statistically validated repeatability metrics.

### 3.7. Apparent Thermal Footprint of a Human-Sized Target at Different Heights

**Figure 7:**
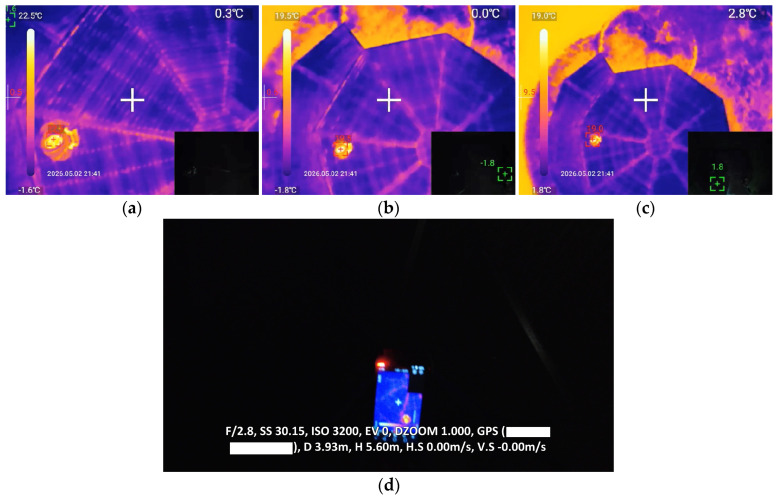
Apparent thermal footprint of a human-sized reference target at different low-altitude operating heights: (**a**) direct UTi260M smartphone-recorded thermal frame at approximately 5.6 m; (**b**) direct UTi260M thermal frame at approximately 10 m; (**c**) direct UTi260M thermal frame at approximately 15 m; (**d**) UAV RGB telemetry reference frame used only to assign the approximate operating height. The direct UTi260M frames were used for qualitative target-size verification, whereas the UAV RGB frame was not used for pixel-size or temperature analysis.

Representative direct UTi260M thermal frames were analyzed to evaluate the apparent thermal footprint of a human-sized target at different low-altitude operating heights, as shown in Figure 7. The selected frames were recorded directly by the Servo King9000 smartphone from the UTi260M thermal camera. The UAV-recorded RGB video was used only to assign the approximate flight height from the telemetry overlay and was not used for pixel-size estimation.

The results show that the human-sized thermal target remained visually distinguishable from the nighttime background at the tested low-altitude heights. At lower height, the target occupied a larger apparent pixel footprint, which improved visual separability. At higher height, the physical target did not emit less heat, but its apparent footprint became smaller in the thermal frame and was more affected by background mixing, limited thermal resolution, motion, and non-radiometric application-level image processing.

Therefore, the field results support the interpretation that the proposed workflow can provide qualitative indication of clearly visible human-sized thermal hotspots under favorable nighttime or low-glare conditions. However, this procedure should not be interpreted as validated automatic human detection, nor as a quantitative thermal measurement method. The corresponding pixel-size-assisted qualitative verification results for the human-sized thermal target are summarized in Table 7.

### 3.8. Stepwise Thermal Screening Protocol

Because the suspended payload reduced endurance and increased motor-region thermal loading, the workflow is more suitable for short, sector-based preliminary screening than for continuous large-area thermal surveys. A practical operating protocol consists of short flights to a selected sector, brief visual thermal observation, return before critical battery depletion, battery replacement, short motor cooling, system reset, and deployment over the next sector.

The recommended stepwise thermal screening protocol is summarized in Table 8.

This protocol is proposed only as a practical operational recommendation for the tested low-cost configuration. It should not be interpreted as a validated search-and-rescue procedure or as a replacement for professional UAV thermal mapping workflows.

### 3.9. Dual-Channel Verification of Thermal Observations

Representative qualitative comparisons between the direct UTi260M thermal output and the UAV-recorded smartphone display are presented in Figure 6. The direct thermal frames provided clearer thermal boundaries and better visual separation of the hotspot from the background. The UAV RGB frames showed the same thermal information indirectly through the smartphone display, but with reduced sharpness and additional display-related limitations.

Although the suspended smartphone module exhibited visible oscillation during flight, clear thermal hotspots remained visually distinguishable in selected nighttime flight segments. However, the UAV-recorded frames should be interpreted only as indirect visual confirmation of the smartphone-displayed thermal stream. They are not radiometric thermal images and cannot be used for quantitative temperature measurement or accurate shape delineation.

Overall, the results from Section 3.5, Section 3.6, Section 3.7 and Section 3.8 show that the proposed workflow can support qualitative visual indication of clear thermal hotspots under favorable low-glare or nighttime conditions. Its usability is limited by display readability, payload oscillation, altitude-dependent display size, RGB camera exposure, and the non-radiometric nature of the indirect display recording.

### 3.10. Thermal Loading of UAV Motors Under Loaded and Unloaded Conditions

**Figure 8:**
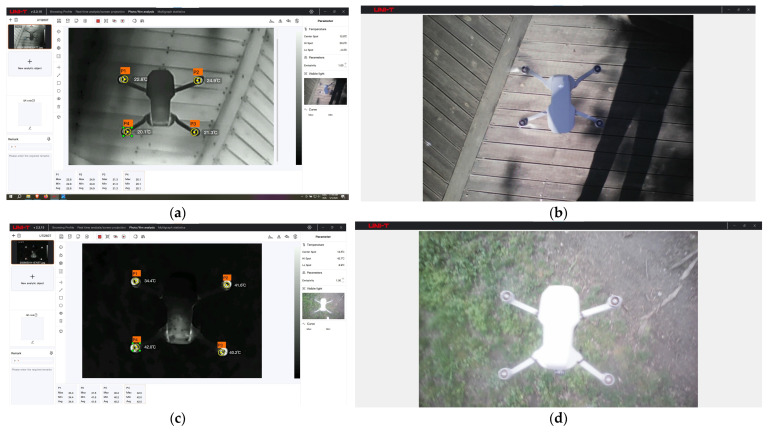
Comparison of UAV motor-region thermal response under loaded and unloaded operating conditions: (**a**) visible-light reference image of the UAV under loaded operation; (**b**) thermal analysis of the UAV motor regions under loaded operation, showing increased motor-region temperatures and non-uniform thermal distribution; (**c**) visible-light reference image of the UAV under unloaded operation; (**d**) thermal analysis of the UAV motor regions under unloaded operation, showing lower and more balanced motor-region temperatures. Measurement points P1–P4 correspond to the four motor locations used for comparative thermal assessment. The point-based motor-region temperatures are summarized in Table 9.

To evaluate the effect of payload on motor thermal loading, motor-region thermal observations were performed under loaded and unloaded operating conditions. Figure 8 compares the motor-region thermal distributions for both cases. Under loaded operation, the recorded temperatures were higher and more spatially non-uniform, indicating increased motor loading. In contrast, the unloaded configuration showed lower and more balanced temperatures across the four motor regions.

The point-based values extracted from the four motor regions were further used to calculate the maximum motor temperature and motor thermal asymmetry according to Equations (8) and (9).

**Table 9:** Comparative motor-region thermal observations under loaded and unloaded operating conditions.

Motor	With Payload (°C)	Without Payload (°C)	Difference (°C)
P1	34.4	22.8	11.6
P2	41.6	24.9	16.7
P3	40.2	21.3	18.9
P4	42.0	20.1	21.9

The higher loaded motor-region temperatures were not spatially uniform across the four motor locations. This non-uniformity was attributed to the combined effect of suspended-payload alignment, possible center-of-gravity offset, payload oscillation, rotor downwash interaction with the suspended module, and local differences in propulsion demand during stabilization. Therefore, the motor-region results should be interpreted as practical indicators of increased propulsion loading rather than as a calibrated thermal diagnosis of individual motor performance.

Using Equation (9), the motor thermal asymmetry increased from 4.8 °C under unloaded operation to 7.6 °C under loaded operation. In addition, the maximum observed motor-region temperature increased from 24.9 °C to 42.0 °C. The point-based loaded–unloaded differences ranged from 11.6 °C to 21.9 °C, indicating a clear payload-induced increase in motor-region thermal loading. These results support the interpretation that the suspended payload increased propulsion demand and produced a more non-uniform motor-region thermal distribution under loaded operation.

The non-uniform thermal distribution is consistent with the suspended payload configuration, possible center-of-gravity offset, airflow interaction, and the non-rigid mounting arrangement.

### 3.11. Payload-Induced Propeller Warning and Operational Failure Mode

**Figure 9:**
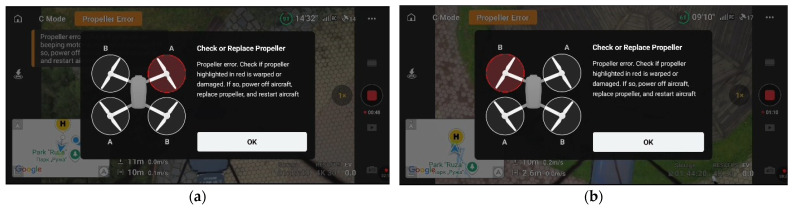
Operational warnings observed during payload flights: (**a**,**b**) representative “Propeller Error” warnings associated with payload operation, center-of-gravity sensitivity, and increased propulsion loading.

During payload flights, the UAV occasionally displayed a “Propeller Error” warning, as shown in Figure 9. The warning was not observed during the no-payload baseline flights. The warning occurrence was associated with payload operation, especially when the suspended smartphone–thermal camera module was not ideally centered or was slightly inclined toward the front motors.

### 3.12. Exploratory Operational Relationship Analysis

**Figure 10:**
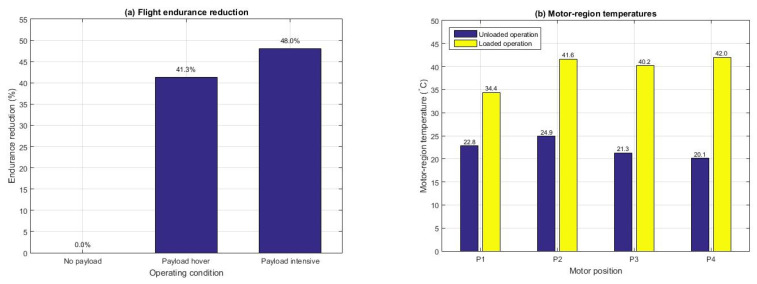

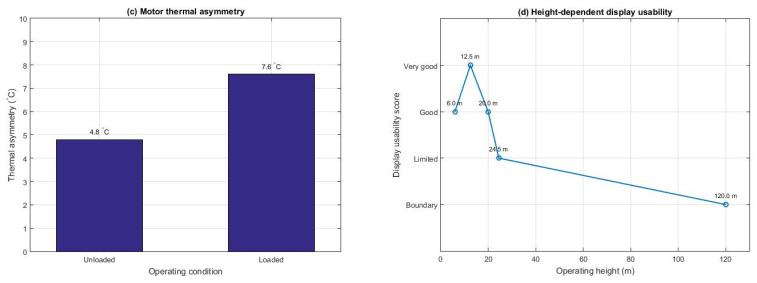
Exploratory operational relationships of the proposed UAV thermal sensing workflow: (**a**) flight endurance reduction under no-payload, payload-hover, and intensive payload-flight conditions; (**b**) motor-region temperatures at P1–P4 under unloaded and loaded operating conditions; (**c**) motor thermal asymmetry under unloaded and loaded operation; (**d**) relationship between operating height and display usability score. The plots summarize the main observed tendencies: payload operation reduced endurance, increased the maximum observed motor-region temperature from 24.9 °C to 42.0 °C, increased motor thermal asymmetry from 4.8 °C to 7.6 °C, and confirmed the best display usability in the 10–20 m operating range.

To summarize the main operational relationships observed during the experiments, selected descriptive plots were prepared from the measured endurance, motor-temperature, motor-asymmetry, and image-usability data. Because of the limited number of repeated trials, these plots were interpreted as exploratory operational relationships rather than statistically confirmatory correlations. The resulting exploratory operational plots are shown in Figure 10.

### 3.13. Descriptive Entropy Analysis of Smartphone-Displayed Thermal Frames

**Figure 11:**
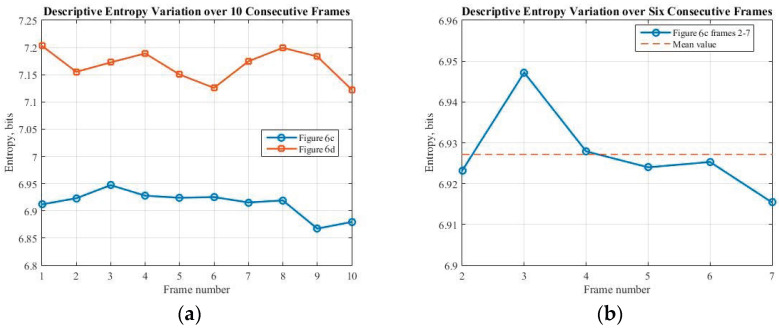
Descriptive entropy analysis of smartphone-displayed thermal frames: (**a**) entropy variation over six selected consecutive frames from the scene shown in Figure 6c; (**b**) entropy variation over ten consecutive frames from the scenes shown in Figure 6c,d. Entropy was calculated from identical 8-bit grayscale regions of interest for each frame sequence. The plots are presented only as relative image-information indicators and should not be interpreted as radiometric or statistically validated performance metrics.

A descriptive entropy analysis was performed on smartphone-displayed thermal frames from the scenes shown in Figure 6c,d. The purpose of this analysis was to document short-term frame-to-frame variation in displayed image-information content within selected regions of interest. Figure 11b presents the six selected consecutive frames from Figure 6c, while Figure 11a provides an additional comparison over ten consecutive frames from Figure 6c,d. The analysis is descriptive and is not intended as radiometric validation or formal statistical repeatability assessment.

For the six selected frames from Figure 6c, the calculated entropy values were 6.9232, 6.9472, 6.9279, 6.9240, 6.9253, and 6.9154 bits, respectively. These values remained within a narrow range, with a mean value of 6.9272 bits and a standard deviation of 0.0107 bits. This indicates stable short-term image-information content within the selected region of interest under the tested condition. The additional ten-frame comparison shows that the Figure 6d sequence exhibited higher entropy values than Figure 6c, but this difference should be interpreted only as scene- and display-dependent variation in the recorded thermal visualization, not as improved radiometric quality or detection performance.

## 4. Discussion

### 4.1. Feasibility of the Minimum-Cost Indirect Workflow

The proposed configuration demonstrated that a minimum-cost indirect UAV thermal visualization workflow can be implemented using a lightweight consumer UAV, a compact smartphone, and a smartphone-connected thermal camera. The key enabling element was the Servo King9000 smartphone, which allowed the UTi260M thermal stream to be operated, displayed, and recorded, while the UAV onboard RGB camera captured the smartphone-displayed thermal information during flight.

This result is relevant for educational demonstrations, preliminary field screening, rapid prototyping, and low-resource experimental applications. However, the system should not be interpreted as a professional UAV thermal imaging platform. It is an indirect display-based visualization workflow, not a direct radiometric aerial thermography system. Therefore, its practical value is limited to qualitative visual indication of clear thermal hotspots under favorable operating conditions.

### 4.2. Payload Penalty, Stability, and Propulsion Loading

Although the airborne smartphone–thermal camera module had a mass below 100 g, its relative effect on the sub-250 g UAV platform was substantial. The effective added payload was approximately 91 g, corresponding to about 36.5% of the UAV mass. This payload ratio reduced the available endurance margin and increased the sensitivity of the UAV to payload alignment, suspended-load motion, rotor downwash, and propulsion loading.

The observed endurance reduction from approximately 25 min to approximately 14 min 40 s under near-ground hover conditions confirms that the payload imposed a major energy penalty. The more intensive payload profile resulted in an even shorter usable flight duration of approximately 13 min. Therefore, the configuration is not suitable for long-duration flights or continuous large-area surveys.

The post-flight motor thermal results also indicate that the payload affected propulsion loading. However, the interpretation should be made carefully. The payload configuration increased the maximum post-flight motor-region temperature and substantially increased motor thermal asymmetry, but not every individual motor measurement increased. This is consistent with a suspended and slightly asymmetric payload, where airflow, payload orientation, cooling before measurement, and measurement-point selection may produce non-uniform motor-region temperatures.

### 4.3. Indirect Display-Based Acquisition and Radiometric Limitations

The most important methodological limitation of the workflow is the indirect acquisition chain. The UTi260M thermal camera produced the primary thermal stream, the Servo King9000 smartphone displayed this stream, and the DJI Mini 4K onboard RGB camera recorded the smartphone display. Therefore, the UAV-recorded output is not thermal data in the radiometric sense, but an RGB video of a display showing thermal information.

This distinction affects the interpretation of all image-based results. The UAV-recorded frames are influenced by display brightness, ambient illumination, reflections, RGB camera exposure, focus, screen size in the frame, compression, suspended-load motion, and acquisition geometry. As a result, the workflow cannot provide quantitative temperature measurement, radiometric thermal mapping, calibrated thermal contrast, or accurate thermal shape delineation.

For this reason, the system should be considered suitable only for qualitative visual confirmation that a clearly distinguishable thermal hotspot is present in the smartphone-displayed stream. Any use beyond this, such as accurate target detection, thermal mapping, or temperature-based diagnostics, would require direct radiometric acquisition, calibrated thermal data, controlled targets, and repeated validation trials.

The descriptive entropy check in Figure 11a confirms short-term stability of the displayed image-information content for the selected six-frame interval, while Figure 11b illustrates that entropy can vary between different smartphone-displayed thermal scenes. However, this does not remove the main limitation of the workflow: the UAV-recorded output remains an RGB recording of a smartphone display rather than direct radiometric thermal data.

### 4.4. Illumination, Display Readability, and Operating Height

The results showed that illumination strongly affected the usability of the indirect UAV recording. Daylight operation was limited by display glare and reflections, while nighttime or low-glare conditions improved the readability of the smartphone-displayed thermal stream. However, excessive smartphone brightness during nighttime operation could cause partial overexposure in the UAV RGB recording. Therefore, display brightness must be adjusted according to the ambient illumination.

The most practical usability was observed at approximately 10–20 m under favorable low-glare or nighttime conditions. At lower heights, payload motion and stabilization effects were more apparent, while at higher heights the smartphone display occupied a smaller part of the UAV RGB frame and became more difficult to interpret. The altitude values reported in this study should not be interpreted as detection thresholds. They describe only the practical readability of the smartphone-displayed thermal stream under the tested conditions.

### 4.5. Operational Window and Practical Use

The proposed workflow is most appropriate for short-duration, stepwise preliminary screening of selected areas under calm and low-glare conditions. It is not suitable for long-range inspection, continuous area mapping, high-speed flight, or missions requiring quantitative thermal data.

A practical use scenario would consist of short flights over predefined sectors, brief visual inspection of the smartphone-displayed thermal stream, return with battery reserve, controlled landing, motor cooling, battery replacement, and repetition over the next sector. This use case is consistent with the limited endurance, suspended-load oscillation, and qualitative nature of the indirect display-based acquisition.

The main findings and their operational implications are summarized in Table 10.

### 4.6. Limitations and Future Work

This study has several limitations. It used one UAV platform, one smartphone–thermal camera configuration, a limited number of field observations, and non-controlled environmental conditions. Wind, ambient temperature, illumination, battery state, and exact flight profiles were not controlled using calibrated field instrumentation. The thermal targets were not defined through a formal ground-truth protocol, and no statistical detection criteria, confidence intervals, or detection probabilities were established.

For this reason, the results should be interpreted as operational feasibility evidence rather than statistically validated detection performance.

The UTi260M data were obtained through a smartphone application rather than through raw radiometric matrices. The UAV RGB recording was an indirect display capture affected by display characteristics, exposure, focus, compression, and payload motion. The motor thermal measurements were acquired after landing and should be treated as conservative indicators rather than real-time in-flight motor temperatures.

Future work should include repeated flights under controlled environmental conditions, defined ground-truth thermal targets, documented target dimensions and distances, battery start–stop rules, wind and ambient-temperature logging, direct radiometric data extraction if supported by the sensor, synchronized video acquisition, improved mechanical alignment, and in-flight motor thermal telemetry or embedded temperature sensing. A more capable UAV platform and a lightweight rigid mount could also improve repeatability, but such modifications would move the system away from the minimum-cost boundary evaluated in the present study.

## 5. Conclusions

This study evaluated the feasibility and operational limits of a minimum-cost indirect UAV thermal visualization workflow based on a DJI Mini 4K consumer drone, a Servo King9000 lightweight smartphone, and a UTi260M smartphone-connected infrared thermal camera. The system was intentionally designed as a low-cost exploratory configuration and not as a professional radiometric UAV thermal imaging platform.

The results showed that the DJI Mini 4K was able to lift the suspended Servo King9000 + UTi260M module under the tested conditions. The complete UAV–payload configuration had a measured mass of approximately 340 g, corresponding to an effective added payload of 91 g and a payload-to-UAV mass ratio of approximately 36.5%. This payload ratio was substantial for a lightweight consumer UAV and produced clear operational penalties.

Payload operation reduced near-ground hover endurance from approximately 25 min to approximately 14 min 40 s, corresponding to an approximate reduction of 41.3%. Under a more intensive payload flight profile, usable endurance decreased to approximately 13 min, corresponding to an approximate reduction of 48.0%. These results indicate that the workflow is suitable only for short-duration and sector-based preliminary screening rather than continuous large-area operation.

Post-flight motor thermal inspection indicated that payload operation increased the maximum motor-region temperature and strongly increased motor thermal asymmetry. However, the temperature response was not uniform across all measured motor regions. Therefore, the motor results should be interpreted as conservative post-flight indicators of payload-induced propulsion loading rather than as real-time in-flight motor temperature measurements.

The indirect UAV-recorded video was an RGB recording of the smartphone display and not direct thermal data. Therefore, the workflow cannot be used for quantitative temperature measurement, radiometric thermal mapping, calibrated thermal contrast analysis, or accurate thermal shape delineation. Its practical value is limited to qualitative visual indication of clear thermal hotspots under favorable low-glare or nighttime conditions.

The most practical image usability was observed at approximately 10–20 m under the tested low-glare or nighttime conditions. This range should not be interpreted as a validated detection threshold, but only as a practical operating window for display readability and indirect visual assessment in the tested configuration.

Overall, the proposed workflow can be considered feasible as a minimum-cost proof-of-concept for short-range qualitative thermal screening. Its main limitations are payload mass, reduced endurance, suspended-load oscillation, smartphone display readability, sensitivity to payload alignment, post-flight motor thermal loading, and the absence of raw radiometric data.

Future work should include repeated flights under controlled environmental conditions, defined ground-truth thermal targets, documented target dimensions and distances, battery start–stop rules, wind and ambient-temperature logging, improved payload mounting, in-flight motor thermal telemetry or embedded temperature sensing, and direct radiometric data extraction if supported by the thermal camera hardware and software.

## Figures and Tables

**Table 1:** Main technical characteristics and functional role of the experimental components.

Component	Main Characteristics Relevant to the Study	Role in the Workflow
DJI Mini 4K UAV (manufactured in Shenzhen, China)	Lightweight consumer UAV; sub-250 g class; onboard RGB camera; telemetry overlay; limited payload capability	Carrier platform and indirect RGB recording of the smartphone-displayed thermal stream
Servo King9000 smartphone	Compact Android smartphone used as the central display and recording interface for the UTi260M thermal camera; small display; lightweight body; Type-C interface; Android application compatibility; measured together with the UTi260M as part of the airborne thermal-display module	Operates the UTi260M application, displays the infrared video, records the direct thermal stream, and provides the display recorded indirectly by the UAV RGB camera
UTi260M thermal camera	Smartphone-connected LWIR thermal camera; 256 × 192 thermal resolution; application-based image and video acquisition; no raw radiometric matrix export in the tested configuration	Primary thermal sensing unit mounted with the smartphone
Ulefone Armor 27T smartphone	Rugged smartphone used with the UAV controller	UAV control, live monitoring, telemetry visualization, and screen recording
UTi260T handheld thermal camera	Handheld infrared thermal camera used for still thermal inspection and point-based temperature reading	Motor-region thermal observation under unloaded and loaded operating conditions
Cord-based suspension	Ultralight non-rigid suspension with approximately 0.30 m camera–display distance	Mechanical connection between UAV and smartphone–thermal camera module
Digital scale	Mass measurement of UAV and payload configurations	Quantification of payload mass and payload-to-UAV ratio

**Table 2:** Measured mass characteristics of the experimental UAV–payload configuration.

Parameter	Measured Value
DJI Mini 4K mass	249 g
Servo King9000 + UTi260M mass	95 g
Complete UAV–payload mass	340 g
Effective added payload	91 g
Payload-to-UAV mass ratio	36.5%

**Table 3:** Experimental campaigns and measurement scenarios used in the study.

Scenario	Configuration	Number of Observations/Trials	Illumination/Environment
No-payload baseline flight	DJI Mini 4K without suspended module	One documented endurance baseline and additional visual checks	Outdoor field condition; ambient conditions not instrumentally controlled
Payload hover flight	DJI Mini 4K with Servo King9000 + UTi260M suspended module	One documented endurance test and repeated short operational checks	Outdoor field condition; payload exposed to rotor downwash
Daylight display recording	Payload configuration with UAV RGB camera recording the smartphone display	Representative daylight observations	Daylight; reflections and screen glare present
Nighttime display recording	Payload configuration with UAV RGB camera recording the smartphone display	Representative nighttime observations	Low-light/nighttime condition
Altitude usability assessment	Payload configuration at different heights	Representative observations at selected heights	Mainly nighttime or low-glare condition
Direct thermal recording	UTi260M connected to Servo King9000	Representative direct recordings	Independent of UAV RGB recording; application-based acquisition
UAV-recorded display comparison	DJI Mini 4K RGB camera recording the Servo King9000 display	Representative paired observations	Daylight and nighttime comparison
Motor-region thermal observation	UTi260T observation of UAV motor regions under unloaded and loaded operation	Selected unloaded and loaded motor-region observations	Near-ground/operational field condition
Warning observation	Payload operation under non-ideal alignment and reduced battery margin	Observed during selected payload flights	Outdoor field condition
Human-sized reference thermal target	Direct UTi260M smartphone-recorded thermal frames at selected UAV heights	Representative observations at approximately 5.6 m, 10 m, 11 m, and 15 m	Nighttime/low-background thermal condition
Vehicle-sized reference thermal target	Direct UTi260M smartphone-recorded thermal frames at selected UAV heights	Representative observations of a vehicle-sized thermal object	Nighttime/low-glare field condition

**Table 4:** Flight endurance under no-payload and payload conditions.

Configuration	Flight Mode	Approximate Endurance	Relative Reduction
No payload	near-ground hover	~25 min	—
Payload	near-ground hover	~14 min 40 s	~41.3%
Payload	intensive profile with ascent to 120 m	~13 min	~48%

**Table 5:** Representative motor thermal loading comparison under no-payload and payload conditions.

Condition	Representative Maximum Motor-Region Temperature	Observation
No payload	~23.5 °C	Normal behavior, no warnings
Payload	~42.2 °C	Increased motor loading
Difference	~18.7–20 °C	Payload-induced thermal penalty

**Table 6:** Observed image usability by operating height.

Operating Height	Observed Image Usability	Interpretation
5–7 m	Good	Low-altitude stabilization range; display content remained readable, but payload motion was still visible
10–15 m	Very good	Most practical range for indirect display-based visualization under low-glare/nighttime conditions
20 m	Good	Clear thermal hotspots remained visually distinguishable in the smartphone-displayed stream
24–25 m	Limited to usable	Upper practical range for display readability; the smartphone screen appeared smaller in the UAV RGB frame
120 m	Boundary/stress test	Not recommended as normal operating mode; used only to evaluate the operational boundary of the configuration

**Table 7:** Pixel-size-assisted qualitative verification of a human-sized thermal target.

Target	Approx. UAV Height	Bounding Box	Bounding-Box Area	Relative Footprint	Interpretation
Human-sized thermal target	5.6 m	106 × 65 px	6890 px^2^	100%	Clearly visible
Human-sized thermal target	10 m	49 × 28 px	1372 px^2^	19.9%	Visible, reduced footprint
Human-sized thermal target	15 m	30 × 22 px	660 px^2^	9.6%	Smaller but still distinguishable

**Table 8:** Recommended stepwise thermal screening protocol.

Step	Purpose
Take-off and stabilization	Allow the suspended payload oscillation to settle
Short transit to sector	Avoid high-speed flight and excessive payload swing
Visual thermal observation	Record the direct thermal stream and the UAV display capture
Return with reserve	Prevent critical battery depletion
Controlled landing	Avoid abrupt motion and payload swing
Battery replacement	Restore endurance for the next short flight
Short motor cooling	Reduce accumulated propulsion heat between flights
Next sector	Repeat the procedure over the next selected area

**Table 10:** Main findings and operational implications.

Observation	Operational Implication
The UAV lifted the suspended smartphone–thermal camera module	The concept is feasible under the tested conditions
The effective payload ratio was approximately 36.5%	The payload is substantial for a sub-250 g UAV
Endurance decreased from approximately 25 min to 14 min 40 s/13 min	The workflow is suitable only for short missions
Maximum post-flight motor-region temperature and thermal asymmetry increased	Payload operation increased propulsion loading, but not uniformly in all motor regions
Warnings were observed during selected payload flights	Payload alignment and propulsion margin are critical
Nighttime and low-glare operation improved display readability	Low-glare conditions are preferred for indirect display capture
The most practical usability was observed around 10–20 m	This range describes display readability, not a validated detection threshold
Suspended-load motion affected image stability	Improved mechanical alignment or mounting would improve usability
The UAV-recorded stream was an RGB recording of a smartphone display	The workflow is non-radiometric and qualitative only

## Data Availability

The original contributions presented in this study are included in the article. Further inquiries can be directed to the corresponding author.

## Abbreviations

The following abbreviations are used in this manuscript:

UAV	Unmanned aerial vehicle
RGB	Red, green, blue
LWIR	Long-wave infrared
ROI	Region of interest
